# Arthroscopic Subscapularis Tendon Repair Using a Transpectoral Viewing Portal

**DOI:** 10.1016/j.eats.2025.103515

**Published:** 2025-03-27

**Authors:** F. Okke Lambers Heerspink, Alexandra M. Leenders, Freek Hollman

**Affiliations:** a Department of Orthopedic Surgery, VieCuri Medical Center, Venlo, the Netherlands; b Department of Orthopedics, Maastricht University Medical Centre+, Maastricht, The Netherlands

## Abstract

The subscapularis is the strongest muscle in the rotator cuff and is essential for shoulder function and stability. Arthroscopic repair of the subscapularis tendon can be challenging as the result of muscle retraction, the proximity of the axillary nerve, and impaired visualization. In some cases, a 70° arthroscope is required to enhance the tendon view. We describe the use of a transpectoral viewing portal to facilitate arthroscopic repair of subscapularis tears.

The subscapularis muscle is the largest and strongest muscle of the rotator cuff and plays a critical role in shoulder function.^1^, ^2^, ^3^, ^4^ The subscapularis muscle internally rotates the humerus and acts as an anterior stabilizer of the glenohumeral joint.^1^^,^^5^ Diagnosis of subscapularis tears relies on patient history, clinical assessment, and imaging. Traumatic events, such as anterior shoulder dislocation, can result in muscle weakness and anterior shoulder pain, which may suggest a tear.^6^ Clinical tests, including the lift-off, belly-press, and bear-hug tests, are commonly used, whereas magnetic resonance imaging remains a valuable diagnostic tool.^4^ However, studies indicate that up to 20% of subscapularis tears are only detected during surgery.^4^^,^^7^ Given the tendency for early retraction and fatty infiltration and the important role in shoulder function, many full-thickness subscapularis tendon tears require surgical intervention.^2^ Small subscapularis tears can be repaired intra-articularly via the posterior portal (Lafosse 1/2).^3^ Larger tears can be more challenging. The anterolateral portal is commonly used for viewing, although it has limited visualization of the lesser tubercle. In such cases, a 70° arthroscope may be required.^8^ This Technical Note describes the use of an additional portal, the transpectoral portal, in arthroscopic subscapularis repair. The transpectoral portal offers an excellent view of the lesser tubercle without requiring a 70° arthroscope. Figures depict the portal’s pathway and relationship to adjacent neurovascular structures in a cadaveric specimen.

## Surgical Technique

In the outpatient clinic, the preoperative evaluation includes a medical history, comprehensive clinical examination, and ultrasound or magnetic resonance imaging. Surgery is conducted with the patient under general anesthesia with an added brachial plexus block. The patient is positioned in a beach-chair setup, with the ipsilateral arm supported by the TRIMANO FORTIS arm holder (Arthrex, Munich, Germany). A diagnostic glenohumeral arthroscopy is performed via a standard posterior portal, using a 30° arthroscope. Under direct visualization, standard anterior and anterolateral portals are created using needle localization.

**Fig 1:**
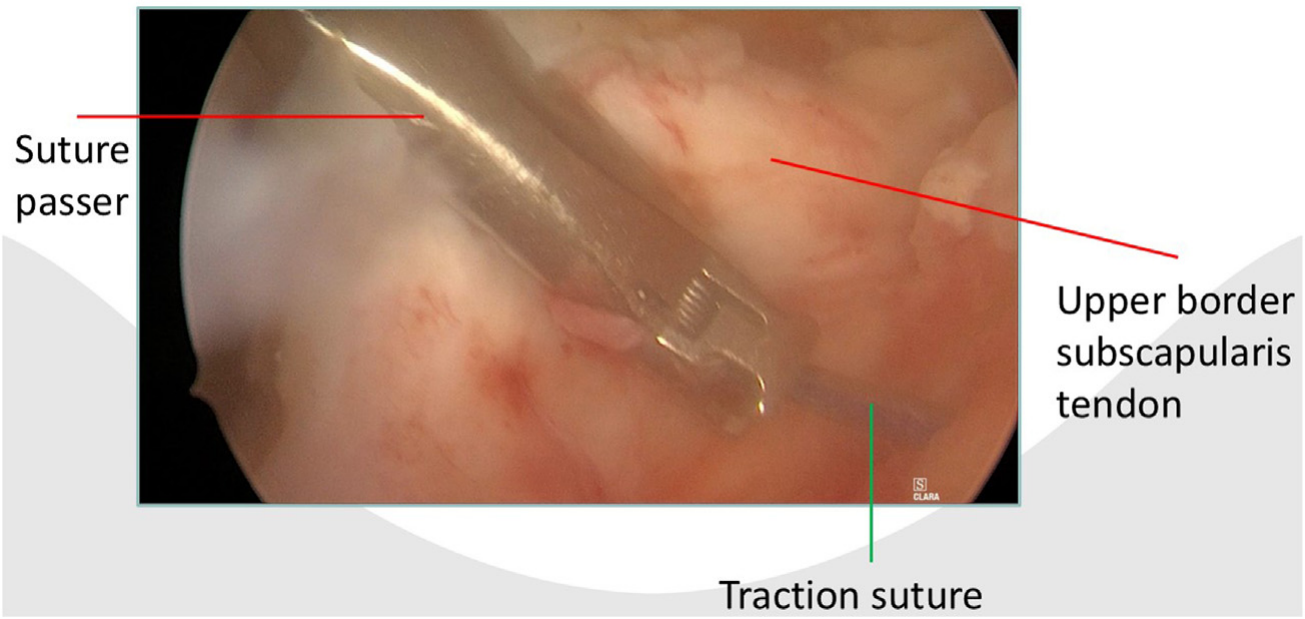
Surgery performed on the left shoulder with the patient in the beach-chair position, with the posterior portal as the viewing portal. Placing a traction suture at the superior border of the subscapularis tendon aids in identifying the superior border and ensures precise suture distribution.

**Fig 2:**
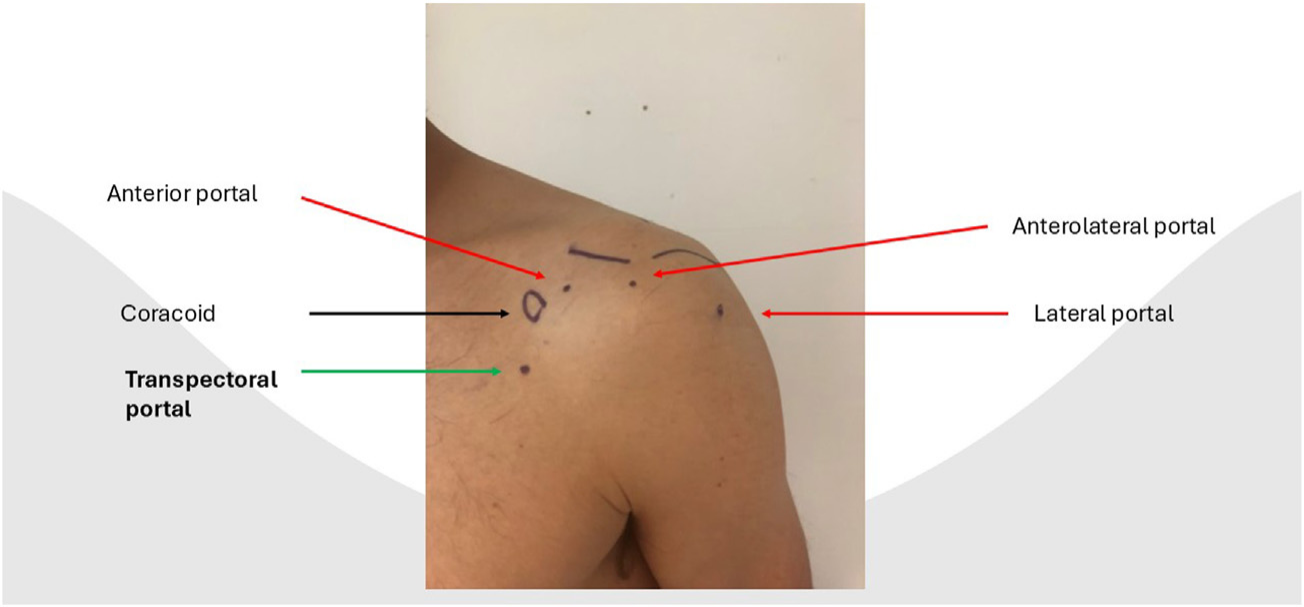
The localization of the different portals used. The transpectoral portal is positioned 2 cm inferior and 1 cm medial to the coracoid process.

**Fig 3:**
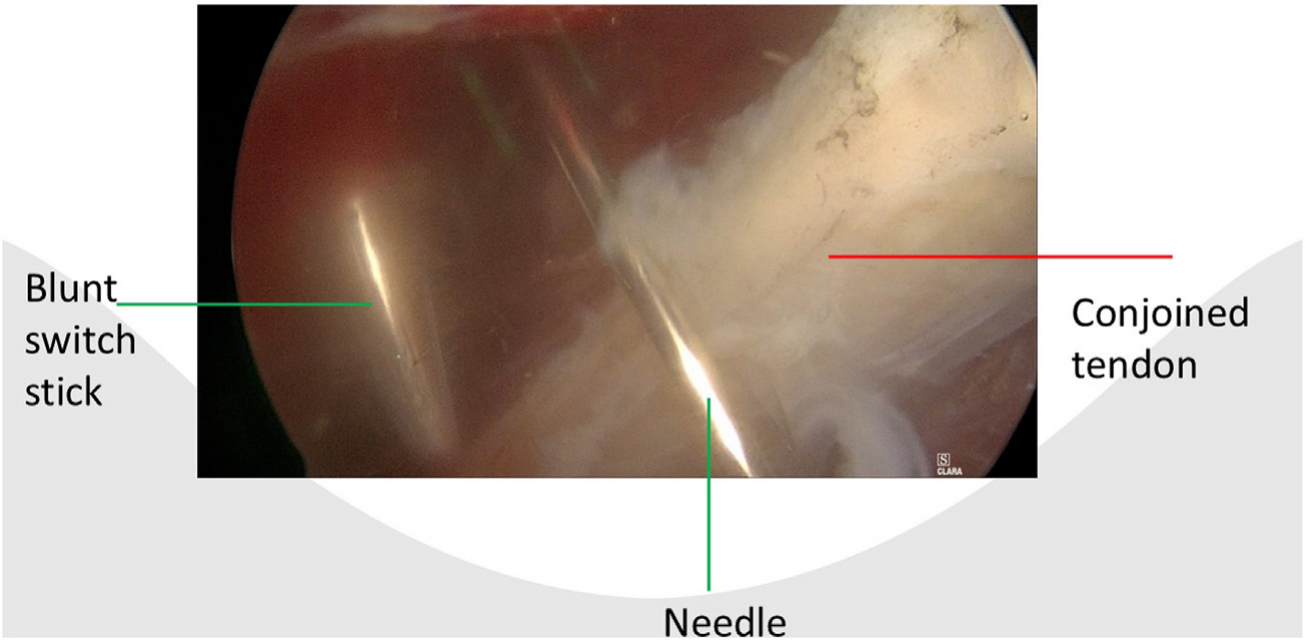
Surgery performed on the left shoulder with the patient in the beach-chair position, with the posterior portal as the viewing portal. The portal is created blunt to avoid damage to neurovascular structures as musculocutaneous nerve.

**Fig 4:**
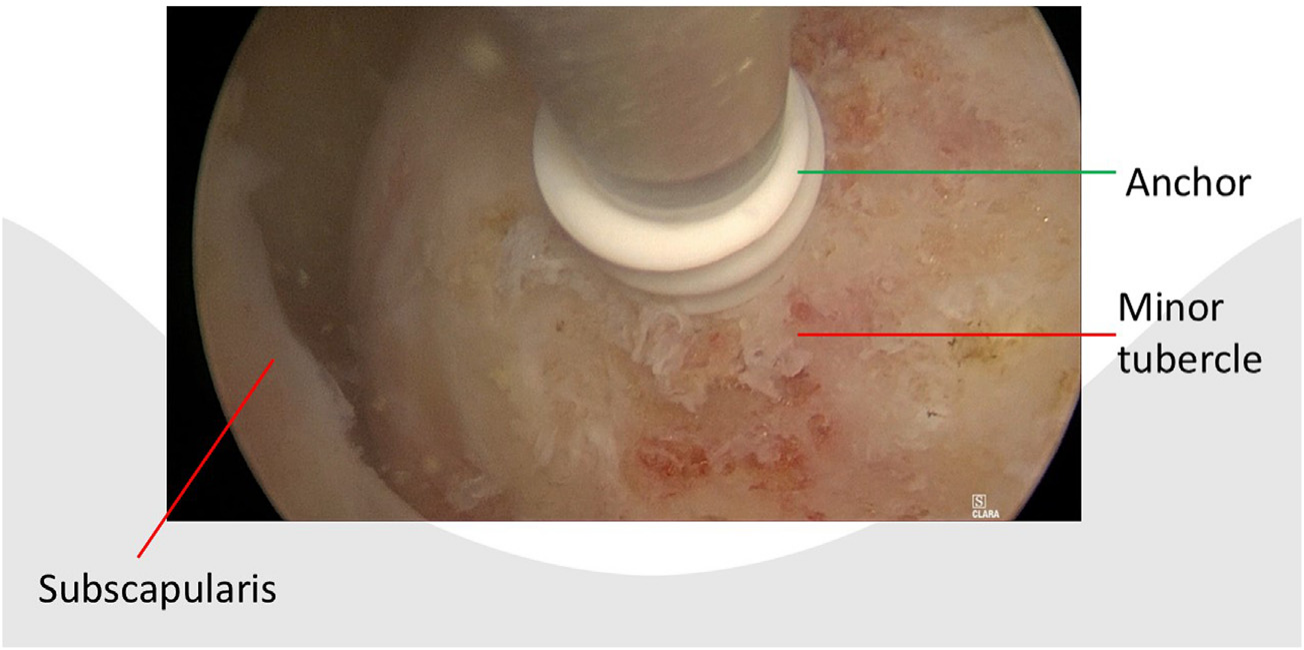
Surgery is performed on the left shoulder with the patient in the beach-chair position, with the transpectoral portal providing an excellent view of the lesser tubercle. An anchor can be inserted through the anterior portal.

**Fig 5:**
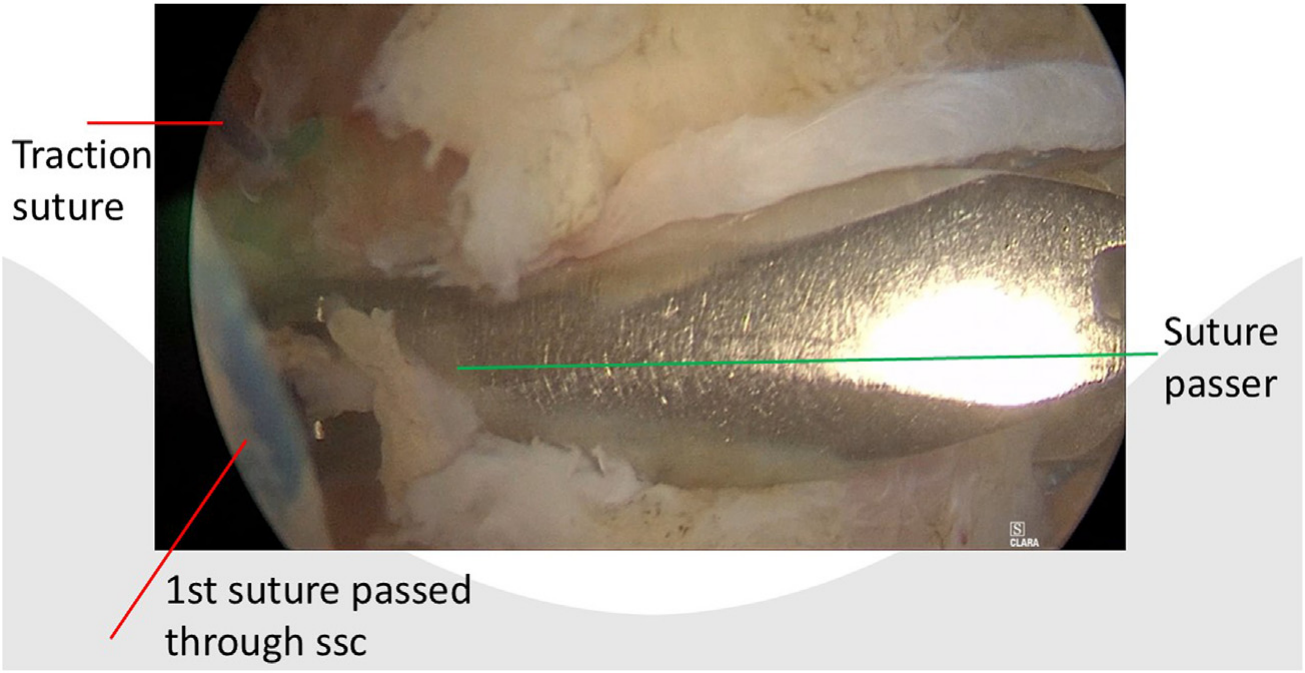
Surgery is performed on the left shoulder with the patient in the beach-chair position, with the transpectoral portal as the viewing portal. The anterior portal is used for suture management, while the suture passer is inserted through the anterolateral portal. The traction suture marks the upper border of the subscapularis tendon.

**Fig 6:**
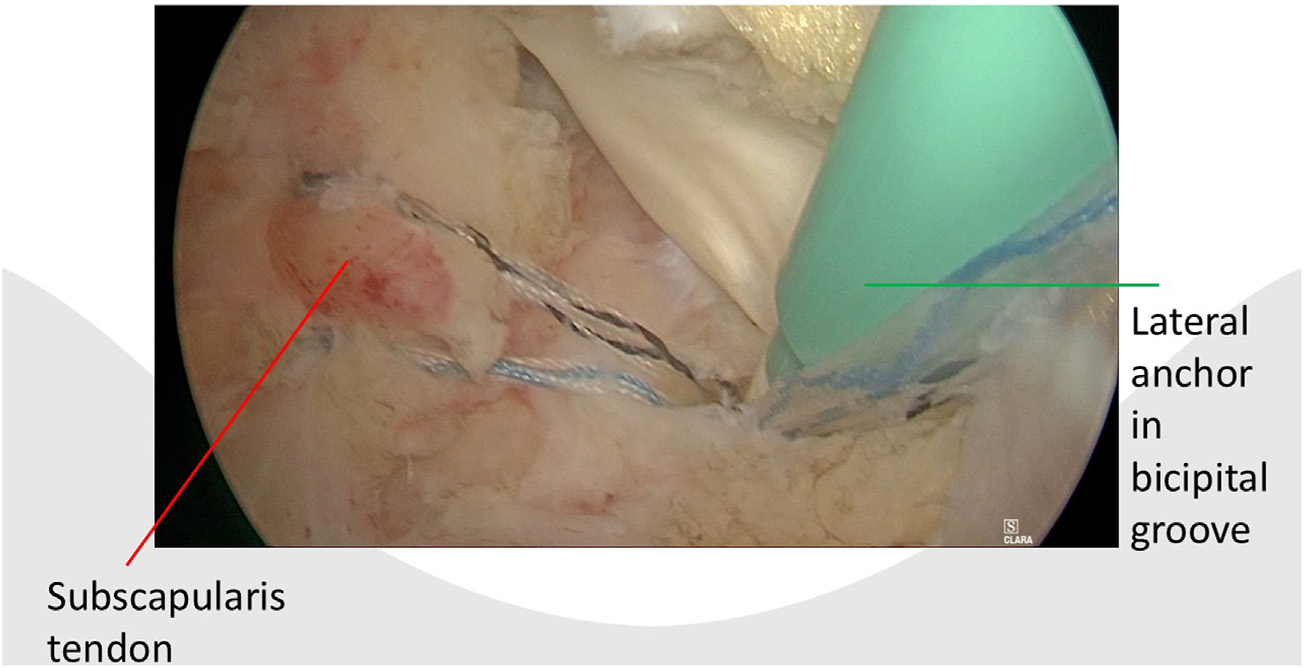
Surgery is performed on the left shoulder with the patient in the beach-chair position, with the transpectoral portal as the viewing portal. Double-row repair of the subscapularis tendon is performed with an lateral anchor in the bicipital groove.

**Fig 7:**
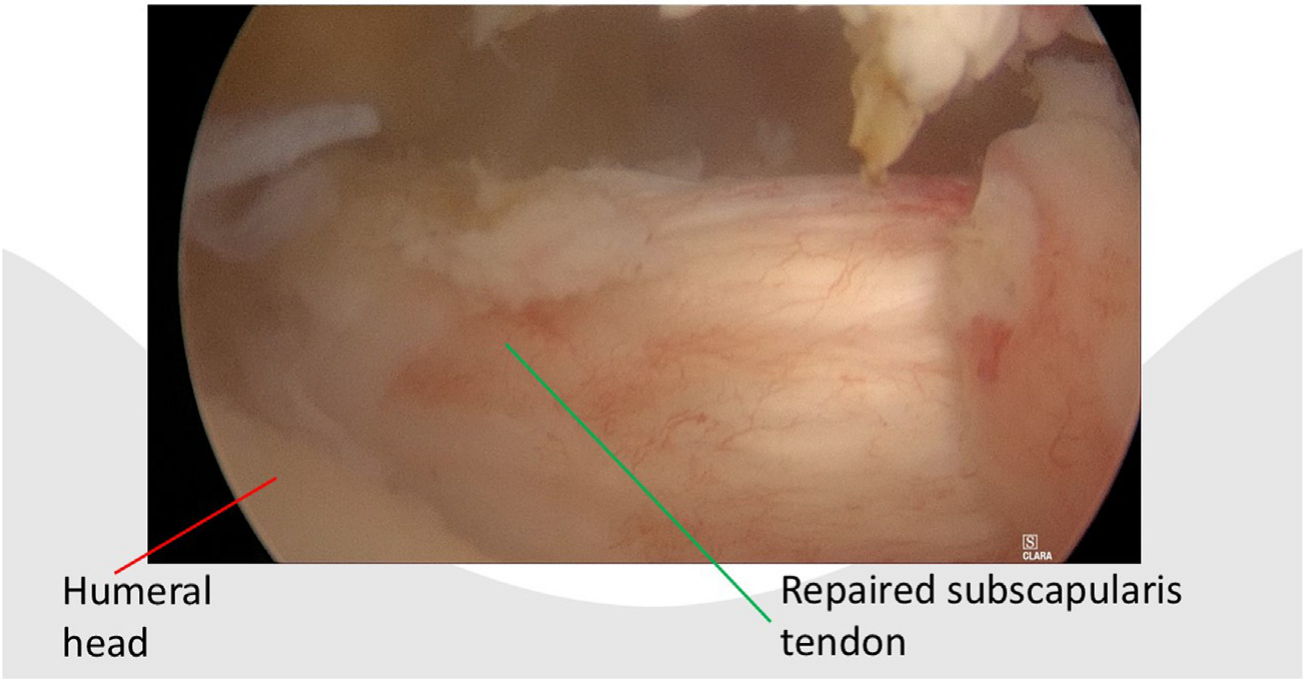
Surgery is performed on the left shoulder with the patient in the beach-chair position, with the posterior portal as viewing portal. Intra articular anatomic repair of a large subscapularis tear after completion of the double-row repair of the subscapularis tendon through the transpectoral portal.

After portal placement, a biceps tenotomy is conducted. Dissection of the transverse ligament proceeds until the subscapularis muscle segment attached to the humeral shaft is reached, preserving the integrity of the J sign when present. A traction suture is placed in the upper border of the subscapularis tendon (Fig 1). This facilitates the view of the subscapularis when passing sutures and prevents sutures being incorporated in the conjoined tendon. In case of a retracted subscapularis tear, a release can be performed through the anterior portal with the posterior or anterolateral portal as viewing portal The transpectoral portal is created under direct visualization, positioned 1 cm medial and 2 cm inferior to the coracoid process, with the needle placed lateral to the conjoint tendon (Fig 2). A blunt switching stick assists in trocar and camera insertion (Fig 3). This portal provides enhanced visualization from the inferior to superior view over the lesser tubercle and serves as the primary viewing portal during repair (Video 1). The footprint is cleared of soft tissue with a burr, and an anchor is inserted at the cartilage edge on the lesser tubercle (Fig 4). Sutures are passed through the subscapularis tendon with a suture passer, tied, and a second anchor is placed in the bicipital groove to complete a double-row repair (Fig 5, Fig 6 and Fig 5, Fig 6). Through the posterior portal a final check is performed on the repaired subscapularis tendon (Fig 7).

## Discussion

**Fig 8:**
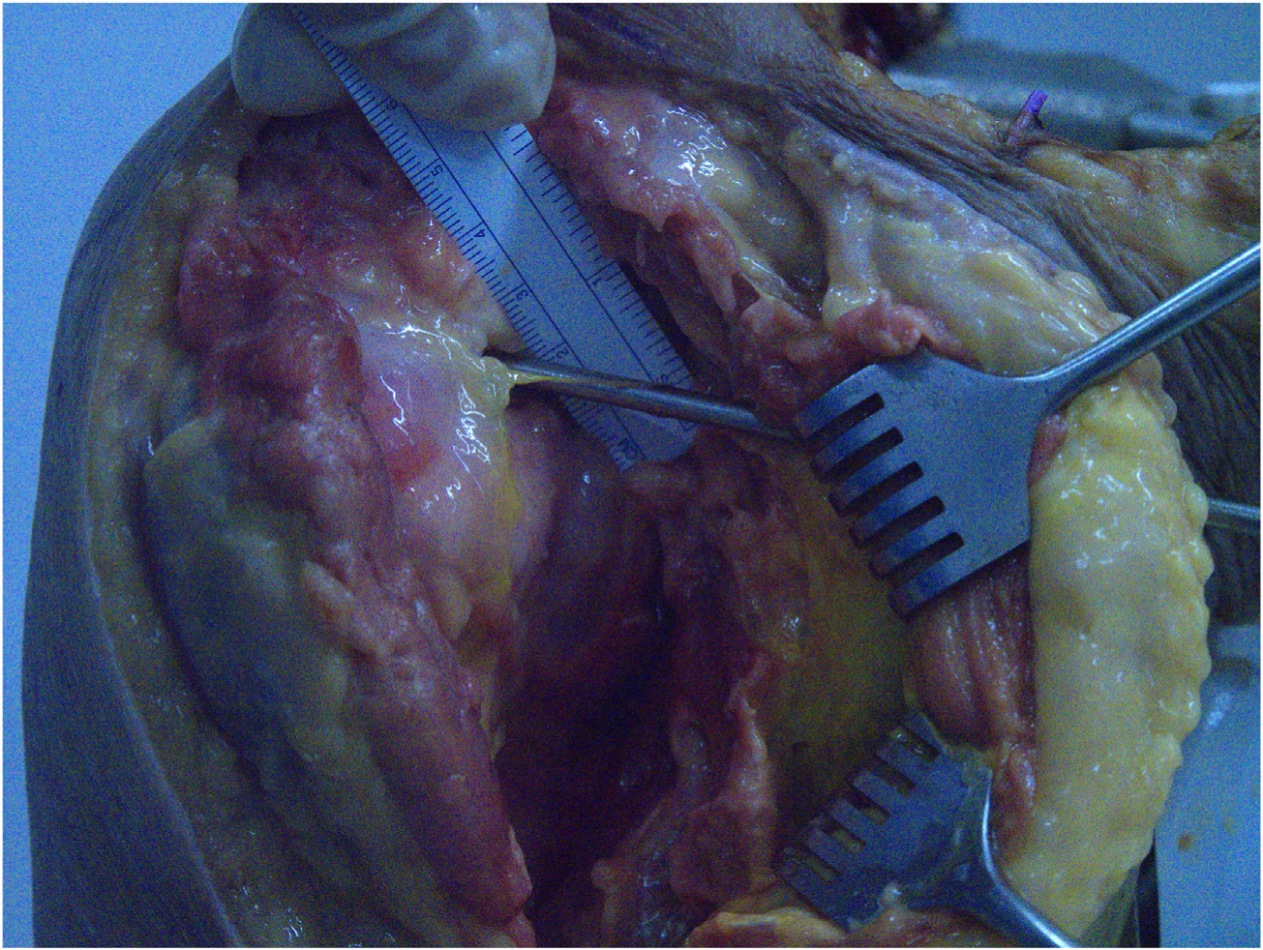
Distance between the deltopectoral portal (indicated by the switching stick) and the musculocutaneous nerve (indicated by the red arrow), in a cadaveric specimen.

The transpectoral portal offers an excellent view of the lesser tubercle without requiring a 70° arthroscope, whereas the standard anterior portal can serve as a working portal. Given that the musculocutaneous nerve pierces the conjoint tendon between 3.1 and 8.2 cm inferior to the coracoid process, this nerve is potentially at risk during portal placement. Careful advancement of the needle and switching stick lateral to the conjoint tendon is essential. To assess the proximity to neurovascular structures, we performed this procedure on a cadaveric specimen. The musculocutaneous nerve was located 2.4 cm from the portal site, which we consider a safe distance (Fig 8).

The transpectoral portal provides an excellent view of the lesser tuberosity and facilitates subscapularis repair, especially in large tears. A traction suture aids in releasing the subscapularis and facilitates suture placement in the tendon, as the upper border is harder to identify compared to the intra-articular approach. This technique also preserves the J-sign, which is beneficial when repairing a concomitant supraspinatus tear. Table 1 list the pros and cons of using the transpectoral portal.

**Table 1:** List of Pros and Cons of Subscapularis Tendon Repair Using the Transpectoral Portal

Pearls	Pitfalls
Locate the transpectoral portal with a needle, approximately 1 cm medial and 2 cm distal to the coracoid process. Incise only the skin, then use a switching stick as a blunt trocar to create the portal.	Although the portal is safely located 2.5 cm from the musculocutaneous nerve, we recommend creating the portal bluntly to minimize the risk of nerve damage.
A traction suture marks the upper border of the SSC, aiding in precise suture placement.	Incorporating the conjoined tendon into the repair.
No need to release the J sign to optimize the view, which facilitates the repair of a concomitant supraspinatus tear	Insufficient release of the transverse ligament limits the inclusion of SSC tissue in the stitches, resulting in an inadequate repair.
Excellent view to perform a double row repair with a lateral anchor in bicipital groove	

SSC, subscapularis.

Lack of experience with arthroscopy in the anteromedial part of the shoulder can pose challenges in achieving clear orientation. A traction suture prevents the conjoined tendon from being incorporated into the repair. Releasing a retracted subscapularis tendon through this portal is not ideal, as the intra-articular portion is not visible. If an insufficient release occurs through the posterior portal, the camera can be switched to the anterolateral portal. A 70° arthroscope is not essential for the procedure; however, the camera must be switched between the posterior, anterolateral and transpectoral portals. For optimal visualization and surgeon posture, the monitor should be positioned behind the patient during the surgical setup. Table 2 discusses the pearls and pitfalls of the transpectoral portal in subscapularis repair. In conclusion, we recommend the transpectoral viewing portal for arthroscopic subscapularis repair, as it provides a direct view of the subscapularis footprint and maintains a safe distance from the musculocutaneous nerve.

**Table 2:** Key Considerations for Subscapularis Tendon Repair Using the Transpectoral Portal: Pearls and Pitfalls

Advantage	Disadvantage
Provides an excellent view for performing a double-row repair with a lateral anchor in the bicipital groove.	Proximity of musculocutaneous nerve.
No need for 70° arthroscope	Switching camera during procedure.
J sign-preserving	Release of the tendon in retracted cases is not possible through the transpectoral portal.
Provides a clear view of the subscapularis, aiding in the precise distribution of sutures.	Lack of experience with arthroscopy in the anteromedial shoulder can make it challenging to achieve and maintain clear orientation.
Release transverse ligament facilitating the potential an additional suprapectoral long head of biceps tendon tenodesis.	Optimize the OR setup to facilitate surgery in the anteromedial shoulder.

OR, operating room.

## Disclosures

All authors (F.O.L.H., A.M.L., F.H.) declare that they have no known competing financial interests or personal relationships that could have appeared to influence the work reported in this paper.

## Supplementary Data

Video 1 In this video, we present a portal for repair of large subscapularis tears. The transpectoral portal offers great visualization over the lesser tubercle and facilitates repair of large subscapularis tears. Surgery is performend on the left shoulder with the patient in beach chair position.
